# Mechanical Coupling Error Suppression Technology for an Improved Decoupled Dual-Mass Micro-Gyroscope

**DOI:** 10.3390/s16040503

**Published:** 2016-04-08

**Authors:** Bo Yang, Xingjun Wang, Yunpeng Deng, Di Hu

**Affiliations:** 1 School of Instrument Science and Engineering, Southeast University, Nanjing 210096, China; wangxingjun2000@126.com (X.W.); dengyunpeng008@gmail.com (Y.D.); di92hu@outlook.com (D.H.); 2 Key Laboratory of Micro-Inertial Instrument and Advanced Navigation Technology, Ministry of Education, Nanjing 210096, China

**Keywords:** decoupled dual-mass micro-gyroscope, quadrature error correction, mechanical coupling error, suppression technology

## Abstract

This paper presents technology for the suppression of the mechanical coupling errors for an improved decoupled dual-mass micro-gyroscope (DDMG). The improved micro-gyroscope structure decreases the moment arm of the drive decoupled torque, which benefits the suppression of the non-ideal decoupled error. Quadrature correction electrodes are added to eliminate the residual quadrature error. The structure principle and the quadrature error suppression means of the DDMG are described in detail. ANSYS software is used to simulate the micro-gyroscope structure to verify the mechanical coupling error suppression effect. Compared with the former structure, simulation results demonstrate that the rotational displacements of the sense frame in the improved structure are substantially suppressed in the drive mode. The improved DDMG structure chip is fabricated by the deep dry silicon on glass (DDSOG) process. The feedback control circuits with quadrature control loops are designed to suppress the residual mechanical coupling error. Finally, the system performance of the DDMG prototype is tested. Compared with the former DDMG, the quadrature error in the improved dual-mass micro-gyroscope is decreased 9.66-fold, and the offset error is decreased 6.36-fold. Compared with the open loop sense, the feedback control circuits with quadrature control loop decrease the bias drift by 20.59-fold and the scale factor non-linearity by 2.81-fold in the ±400°/s range.

## 1. Introduction

The silicon micro-gyroscope, which is a micro-inertial sensor based on micro-electro- mechanical system (MEMS) technology, has undergone 20 years of research and development [1,2,3,4]. The initial silicon micro-gyroscopes that mostly had a single mass cannot distinguish the Coriolis acceleration from the linear acceleration interference in the sensitive axis. To suppress the influence of acceleration or vibration, many researchers have studied the dual-mass silicon micro-gyroscope [5,6,7]. The differential sense capacitors in the dual-mass silicon micro-gyroscopes extract the anti-phase movement of a dual mass in the Coriolis acceleration and suppress the in-phase dual mass movement in the linear acceleration interference. The earlier silicon micro-gyroscopes had coupling structures between the drive mechanism and sense mechanism, which easily led to mechanical coupling between the two modes [8,9]. In [8], the inherent nonlinearity of the parallel plate actuators along the drive and sense axis directions was adopted to tune out the mechanical coupling error resulting from non-ideal stiffness elements. Another previous study [9] capitalized on eight pairs of compensation electrodes to actively compensate the quadrature bias. Simultaneously, many decoupled gyroscopes insulated the drive mechanism from the sense mechanism to reduce the mechanical coupling error [10,11,12]. In [10] two sets of decoupled beams and comb mechanisms were utilized to decouple the drive movement from the sense movement. Similarly, the drive movement in [11] was segregated from the sense movement by three decoupled frames with several decoupled beams. Traditional decoupled frames were simplified as shuttles with decoupled beams to separate the drive movement from sense movement [12]. However, given the difficulty in achieving ideal decoupling beams (the stiffness along the decoupling direction is almost zero, whereas the stiffness in the direction perpendicular to the decoupling direction is infinite), the ideal decoupling performance was almost impossible to implement. The non-ideal decoupling that existed in most decoupled micro-gyroscopes would cause translational movement displacement errors and rotational displacement errors. These non-ideal decoupled errors were sometimes even larger than the other mechanical coupling errors. Simulation results in [13] confirmed that the maximum rotation error displacement of the sense frame derived from the non-ideal decoupling reached 2.7% of the drive displacement in the drive mode.

The present paper reports a mechanical coupling error suppression technology for an improved decoupled dual-mass micro-gyroscope (DDMG). The improved design decreases the moment arm of the drive decoupled torque, which is beneficial for the suppression of the non-ideal decoupled error. Quadrature correction electrodes are added to eliminate further the residual quadrature error. The structure principle, the simulation, and the experiment of the improved DDMG are presented. Section 2 gives an overview of the device description and the quadrature error suppression principle of DDMG. Section 3 presents the structure simulation and fabrication. Section 4 illustrates the quadrature correction and feedback control. Section 5 discusses the experiments. Concluding remarks are finally summarized in Section 6.

## 2. Device Description

### 2.1. Device Principle 

**Figure 1:**
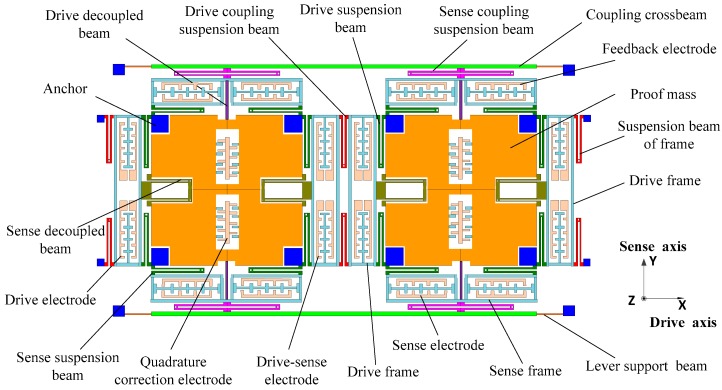
Improved DDMG structural scheme.

Figure 1 shows the improved DDMG structure scheme [14]. The structural parameters under standard atmospheric pressure are shown in Table 1.

**Figure 2:**
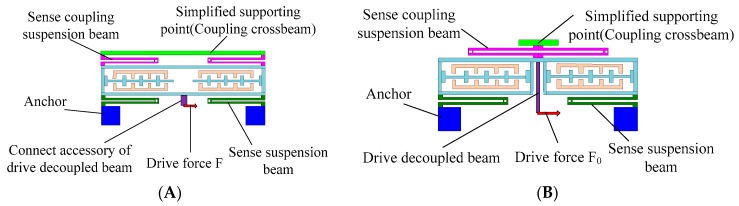
Simplified model of sense frame in the drive mode. (**A**) The original sense frame; (**B**) The improved sense frame.

Utilizing the kinematic coupling of the drive coupling suspension beams, two proof masses realize the in-phase and anti-phase resonance with the same frequency in the drive direction. The resonant frequencies in the drive direction can be calibrated by adjusting the stiffness of the drive suspension beam, drive decoupled beam, and drive coupling suspension beams. Similarly, a complex lever system that is used to achieve the kinematic coupling in the sense direction consists of the coupling crossbeams, sense coupling suspension beams, and lever supporting beams, which ensure the in-phase and anti-phase resonance of two proof masses with the same frequencies in the sense direction. The resonant frequencies can be adjusted by changing the stiffness of the sense suspension beam, sense decoupled beam, and complex lever system. The frequency difference between in-phase mode and anti-phase mode can be added by increasing the coupling rigidity. The increase in the frequency difference reduces the mode coupling between the in-phase mode and anti-phase mode. High modal frequencies are beneficial for suppressing shock and vibration. However, mechanical sensitivity is decreased because of the large modal frequencies. Therefore, the modal distribution should consider a compromise among the mode coupling, mechanical sensitivity, and suppression of shock and vibration. The lever supporting beams can suppress the arc locus at the end of the lever and ensure that the connecting point between the coupling crossbeam and sense coupling suspension beam has a straight trajectory along the sense direction, which is used to remove the rotational motion error of the proof mass in the sense mode.The sense frame and drive decoupled beam are optimized to suppress the mechanical coupling error in the improved DDMG. The simplified model of the sense frame in the drive mode is shown in Figure 2 [14]. The coupling crossbeam is simplified as a supporting point. The connecting point between the drive decoupled beam and the sense frame is moved to the bottom of the improved sense frame, which is utilized to reduce the moment arm of the drive decoupled torque and ultimately remove the drive decoupled torque.

**Figure 3:**
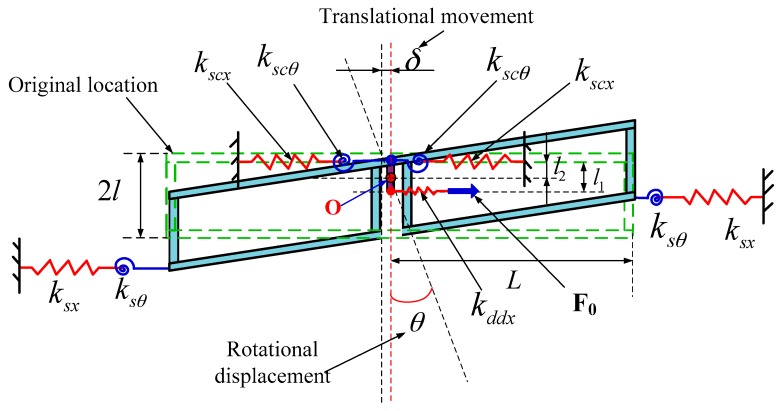
Simplified force model of improved sense frame in the x-axis direction.

The force model of the sense frame in the drive mode shown in Figure 3 is set up to analyze motion characteristics. The drive decoupled beam is simplified as a spring *k*_ddx_ with an equivalent rigid moment arm *l*_1_. δ is the displacement of translational movement. θ is the rotational displacement. *k*_sx_ and *k*_scx_ are the stiffness of sense suspension beam and sense coupling suspension beam along the drive direction, respectively. *k*_sθ_ and *k*_scθ_ are the rotational stiffness of sense suspension beam and sense coupling suspension beam, respectively.

The balance equations are:

(1) $$F_{o}=2k_{scx}(\delta -l_{2}\theta )+2k_{sx}[\delta +(2l-l_{2})\theta ]$$

(2) $$F_{o}(l_{1}-l_{2})=2k_{sx}[\delta +(2l-l_{2})\theta ](2l-l_{2})-2k_{scx}(\delta -l_{2}\theta )l_{2}$$

Solving these equations, then:

(3) $$\delta =\frac{[(2l-l_{1})(2l-l_{2})+2l_{2}^{2}]k_{sx}+l_{1}l_{2}k_{scx}}{8l^{2}k_{scx}k_{sx}}F_{o}$$

(4) $$\theta =\frac{l_{1}k_{scx}+(l_{1}-2l)k_{sx}}{8l^{2}k_{scx}k_{sx}}F_{o}$$

Suppose θ == 0 no matter how the drive force F_o_ changes, then:

(5) $$l_{1}=\frac{2lk_{sx}}{k_{scx}+k_{sx}}$$

Therefore, the rotational displacement can be suppressed by optimizing the drive decoupled beam, sense coupling suspension beam, and sense suspension beam to satisfy Equation (5). When the conditions for Equation (5) are met, then:

(6) $$\delta =\frac{F_{o}}{2(k_{scx}+k_{sx})}$$

(7) $$l_{2}=\frac{2lk_{scx}k_{sx}}{{\left(k_{scx}+k_{sx}\right)}^{2}}$$

### 2.2. Quadrature Error Suppression

**Figure 4:**
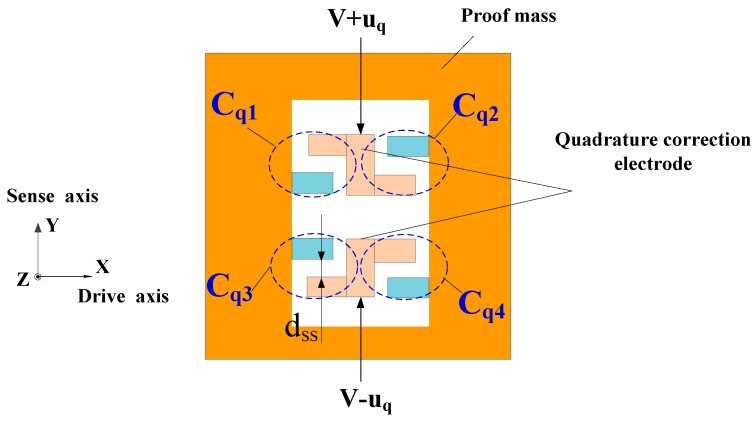
Quadrature error correction scheme.

The quadrature error correction scheme shown in Figure 4 is adopted to restrain further the quadrature error in the DDMG [15]. Four correction capacitors are given by the expressions:

$$C_{q1}\approx n_{q}\frac{\varepsilon_{0}(L_{ss}+x)h}{d_{ss}-y},C_{q2}\approx n_{q}\frac{\varepsilon_{0}(L_{ss}-x)h}{d_{ss}+y},C_{q3}\approx n_{q}\frac{\varepsilon_{0}(L_{ss}+x)h}{d_{ss}+y},C_{q4}\approx n_{q}\frac{\varepsilon_{0}(L_{ss}-x)h}{d_{ss}-y}$$

where *n*_q_ is the number of combs, *L*_ss_ is the overlapping length of the combs, *h* is the thickness of the combs, *d*_ss_ is the comb gap, and *x* and *y* are the displacements along the *X* and *Y* directions, respectively. When the bias voltage *V* and feedback correction *u*_q_ are applied on the quadrature correction electrode, the stiffness matrix of static electric force is:

(8) $$K_{q}\approx \left[\begin{array}{cc} 0 & \frac{4n_{q}\varepsilon_{0}hVu_{q}}{d_{ss}^{2}}\\ \frac{4n_{q}\varepsilon_{0}hVu_{q}}{d_{ss}^{2}} & \frac{4n_{q}\varepsilon_{0}hL_{ss}(V^{2}+u_{q}^{2})}{d_{ss}^{3}} \end{array}\right]$$

where the non-diagonal item stiffness is used to remove the stiffness of quadrature error, and the diagonal item will decrease the sense mode frequency.

## 3. Structure Simulation and Fabrication

**Figure 5:**
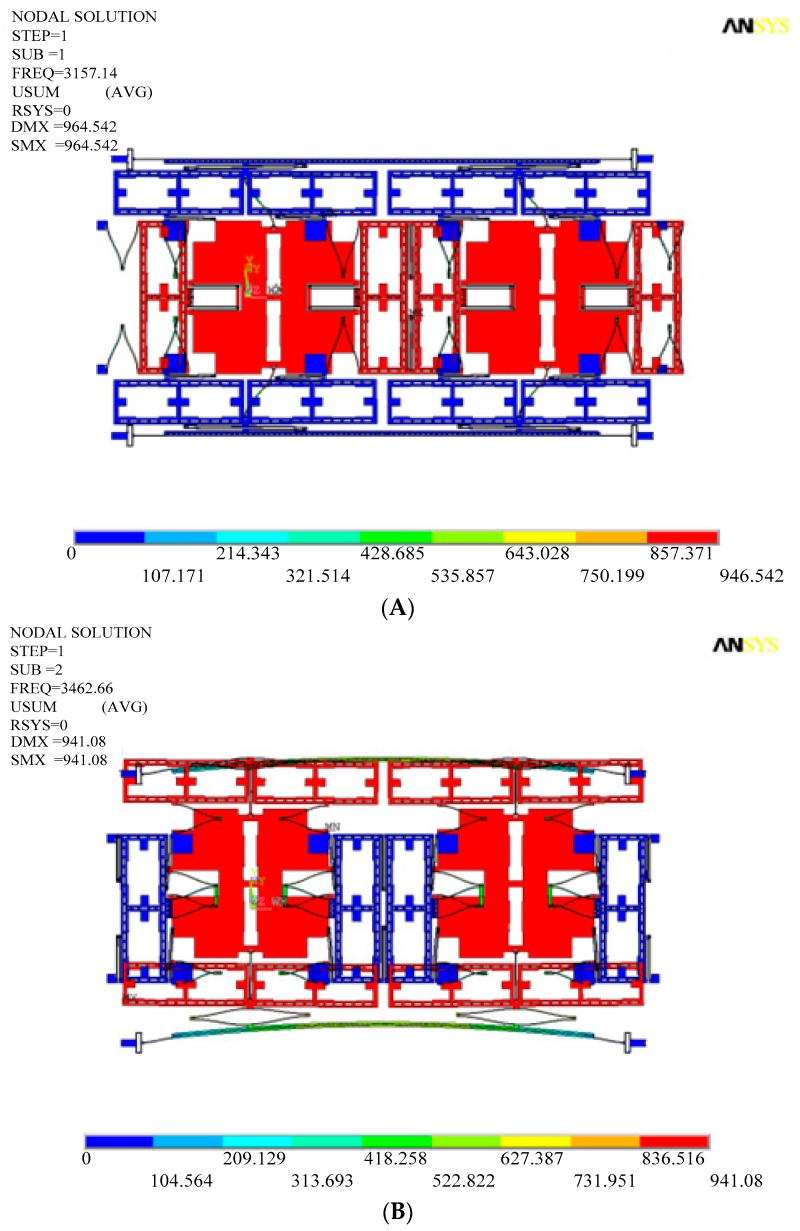

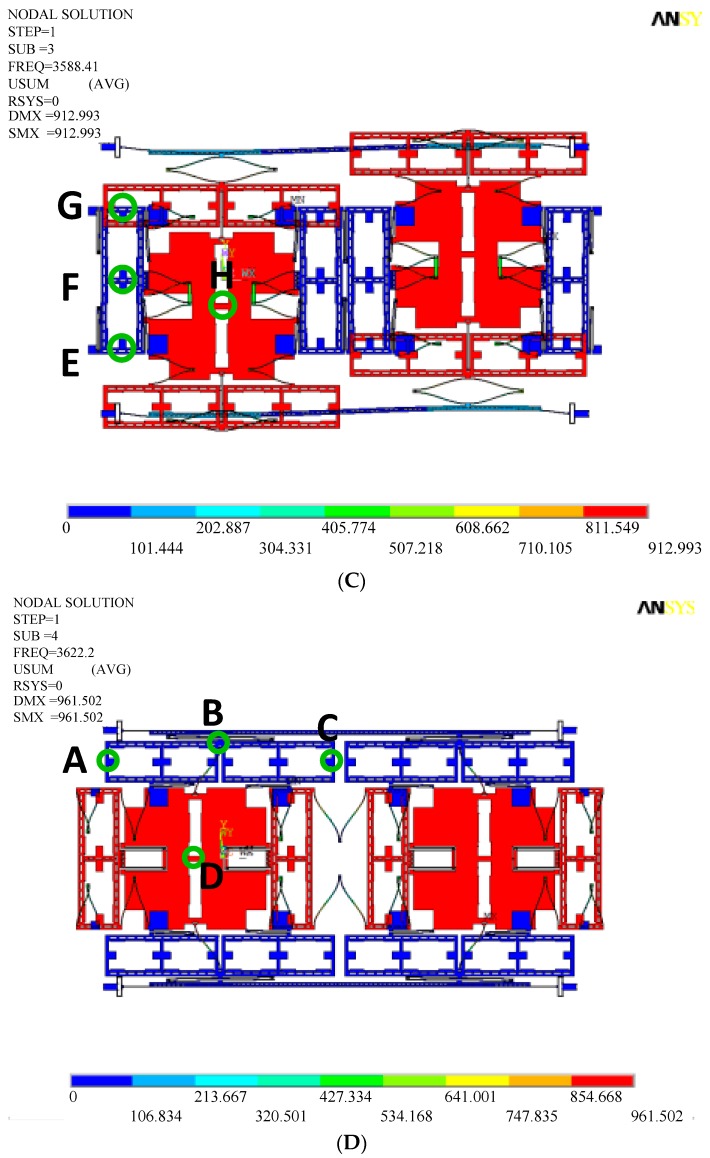
Modal simulation results. (**A**) Drive mode with in-phase movement; (**B**) Sense mode with in-phase movement; (**C**) Sense mode with anti-phase movement; (**D**) Drive mode with anti-phase movement.

The working principle of DDMG was verified by finite element simulation. A finite element simulation model is set up with the ANSYS software. Figure 5 illustrates the simulation results of the drive and sense modes. The first two modes, which are the main interference modes, are in-phase modes in the drive and sense direction and illustrate the acceleration sensitivity. The subsequent two modes, which are main operational modes, are the anti-phase modes in the drive and sense direction and represent the Coriolis sensitivity. The results indicate that the same resonance frequencies of two masses are implemented not only in the drive mode, but also in the sense mode because of kinematic coupling of the coupling beams. In the first fourth modes, the drive mechanism is isolated fully with the sense mechanism, which demonstrates that excellent decoupling performance is achieved. Table 2 presents the frequencies of the first fifth modes of the DDMG. Apparently, the frequencies of the useful third and fourth modes are insulated from those of the other interference modes. In brief, the simulation results prove that the structure principle is feasible.

**Figure 6:**
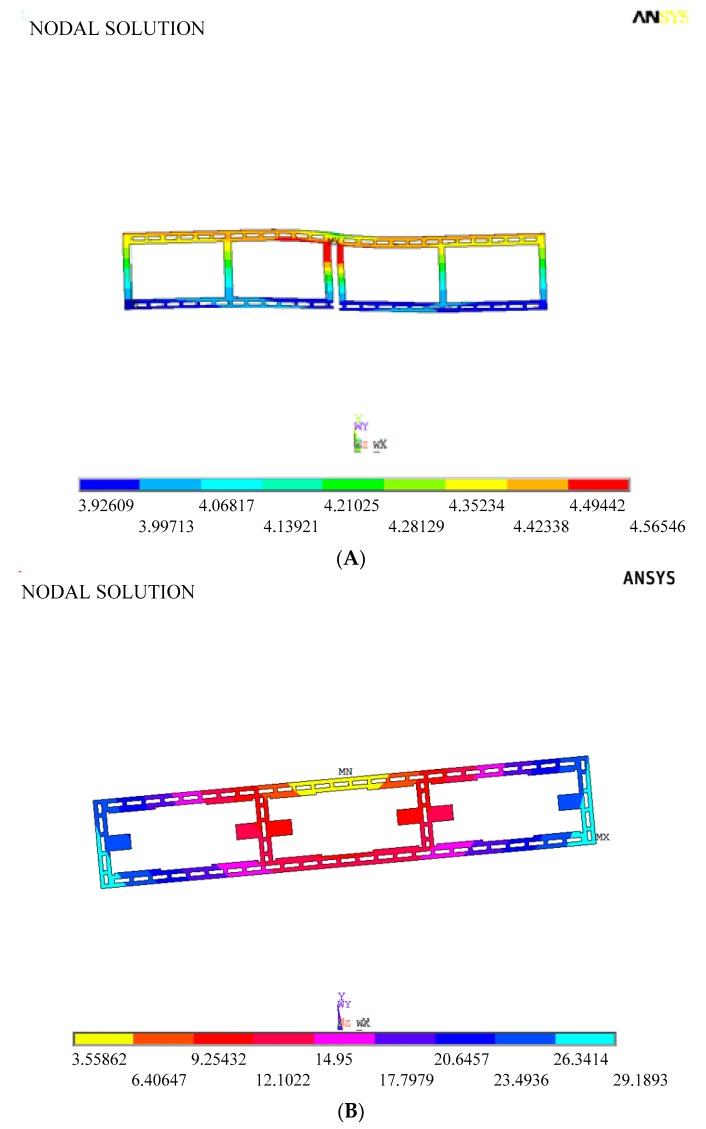
Sense frame displacements in the anti-phase drive mode. (**A**) Improved structure; (**B**) Former structure.

To confirm the suppression effect of the mechanical coupling error in the improved DDSG, the displacements of sense and drive frames are extracted in the drive and sense modes. Figure 6 shows the sense frame displacements in the anti-phase drive mode. Comparing the improved structure with the former structure, the rotational displacements in the improved structure are suppressed significantly by the optimum design. Table 3 shows the non-ideal decoupled displacements in the drive and sense modes (the measured points are shown in Figure 5). Obviously, the translational movement displacements in the improved structure are reduced once in the drive mode (Points A and C displacements along the *x*-axis). Furthermore, the rotational displacements in the improved structure are substantially suppressed in the drive mode (Points A and C displacements along the *y*-axis). This phenomenon is mainly due to the elimination of the drive coupling torque by decreasing the moment arm. Simultaneously, non-ideal decoupled displacements in the sense mode are observed. The translational movement displacements of the drive frame in the improved structure are reduced by 8.2 times in the sense mode (Point E and G displacements along the *y*-axis). The main reason is that suspension beams of the frame are added to increase further the rigidity of the drive frame along the y-direction. Results are consistent with theoretical analysis and demonstrate the feasibility of the optimization design.

**Figure 7:**
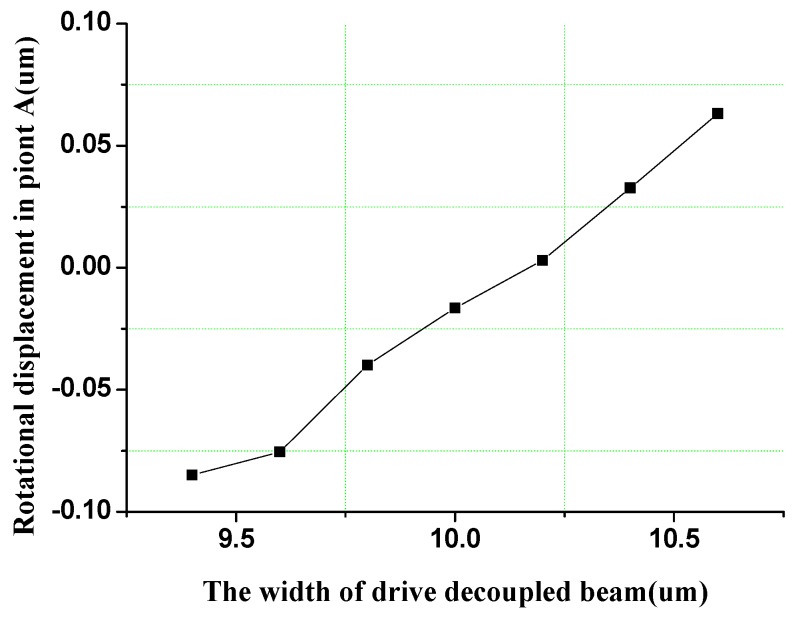
Rotational displacement simulation of sense frame in anti-phase drive mode.

The rotational displacement of the sense frame in the anti-phase drive mode shown in Figure 7 is simulated to verify the influence of the support system stiffness on the rotational movement of sense frame. The rotational displacement can be suppressed completely in the drive decoupled beam with the width of 10 µm. However, the width change in the drive decoupled beam will alter the equivalent rigid moment arm l_1_, so Equation (5) is not satisfied, that is, θ ≠ 0. Obviously, the stiffness variation in the drive decoupled beam results in the emergence of the rotational displacement of sense frame. Moreover, the rotational displacements have an approximate linear relationship with the width of the drive decoupled beams.

**Figure 8:**
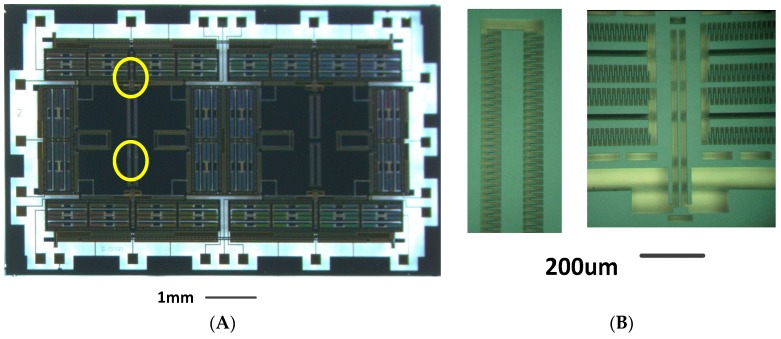
Picture of the structure chip of DDMG. (**A**) Overall structure chip; (**B**) Partial view of the structure chip.

A standard three-mask deep dry silicon on glass (DDSOG) process is adopted to fabricate the structure chip of the DDMG. Figure 8 shows the fabricated structure chip. We adopt a silicon wafer of single crystal with a 4” diameter to fabricate the DDMG device. The fabrication includes the following steps: the photoresist is overlaid on the silicon wafer. The first mask is used to define the bonding area and expose the photoresist. The bonding area is etched by the deep reactive ion etching (DRIE). A Pyrex glass wafer sputters a Cr/Ti/Au layer to establish the electrode wire by the second mask. 

Subsequently, the silicon wafer and the Pyrex glass wafer are connected by the electrostatic anodic bonding process. The thickness of the silicon wafer is decreased by a wet etching process with KOH solution. Finally, the third mask is used to release the silicon structure by the DRIE with 20:1 aspect ratio. Figure 8A shows the overall structure of 8300 μm × 5000 μm × 60 μm. A partial structure shown in Figure 8B includes the drive decoupled beam and quadrature correction combs. The fabricated mechanical structure has a comb gap of 4 µm and a width of suspension beam of 10 µm.

## 4. Quadrature Correction and Feedback Control

**Figure 9:**
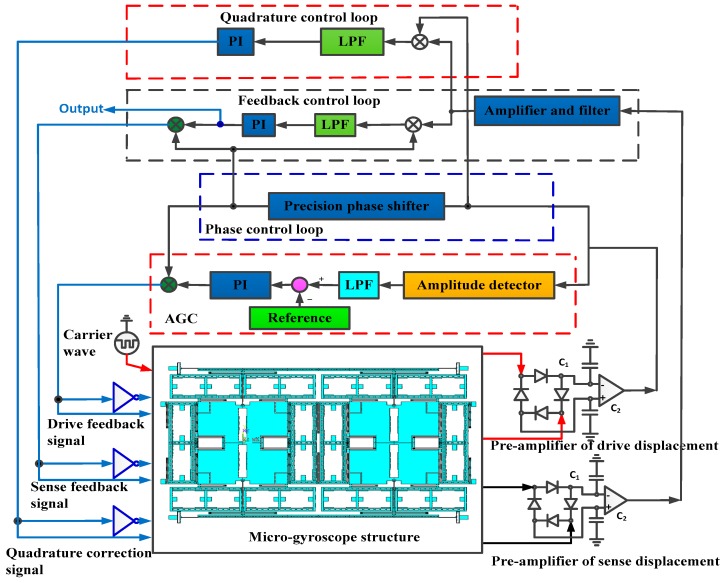
Scheme of the quadrature correction and feedback control circuits.

The scheme of the quadrature correction and feedback control is shown in Figure 9. The circuits include the pre-amplifier, the carrier, the auto gain control (AGC) loop, the phase control loop, the feedback control loop, and the quadrature control loop. 

**Figure 10:**
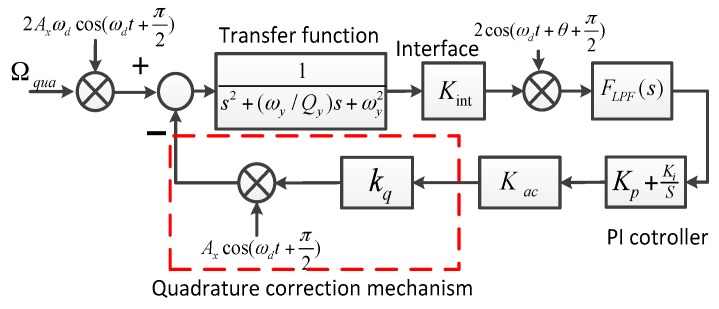
Scheme of the closed-loop quadrature correction control system.

**Figure 11:**
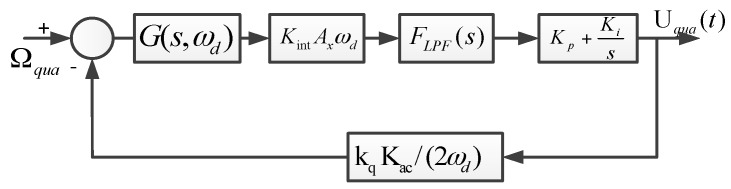
Simplified closed-loop quadrature correction control system.

The AGC loop and phase control loop constitute a closed-loop self-excitation drive circuit. The drive displacement signal is demodulated by the ring diode and magnified by the pre-amplifier. The amplitude of the drive displacement signal is extracted by the amplitude detector and LPF and then contrasted with the reference voltage. The result of the comparator is used to adjust the PI controller output. We utilize the AGC to stabilize the drive displacement amplitude. The phase control loop is designed to satisfy the phase condition of the entire self-excitation drive circuit and locks the natural frequency in the anti-phase drive mode. The total phase shift of the entire self-excitation loop equals 2nπ (where n is an integer). Compared with the quadrature error correction by force balancing [16], the new stiffness cancellation method based on the quadrature correction comb is adopted to suppress the quadrature error. The new quadrature control loop, which consists of the amplifier and filter, demodulation, LPF, and PI, directly feeds back the DC signal to correct the quadrature error, which eliminates the amplitude and phase influence of the modulated AC signal in a force balancing correction manner and improves the quadrature correction accuracy. The feedback control loop, which consists of the amplifier and filter, demodulation, LPF, PI, and demodulation, feeds back the modulated AC signal to compensate the input Coriolis signal. The feedback control loop implements the closed-loop control to suppress the offset error. The scheme of the closed-loop quadrature correction control system is shown in Figure 10. Ω_qua_ is the equivalent angular velocity of the quadrature error. According to Equation (8), *k*_q_ is the stiffness coefficient and *k*_q_ = 4n_q_Ԑ_0_hV/(d_ss_^2^m_y_). m_y_ is the proof mass in the sense mode. F_LPF_(s) is low pass filter. The simplified closed-loop quadrature correction control system is shown in Figure 11.

**Figure 12:**
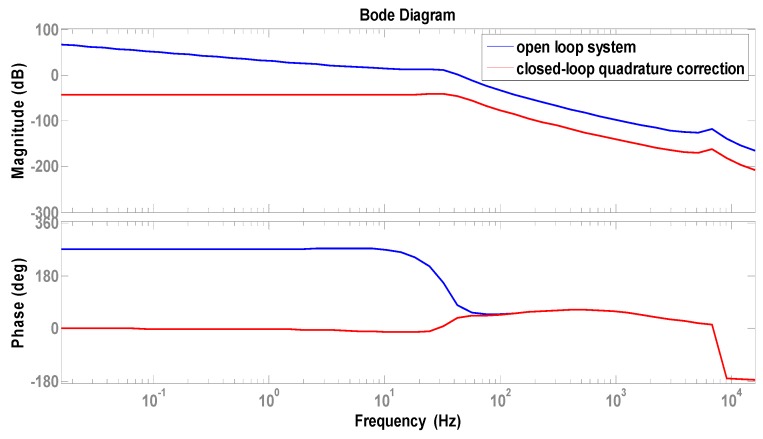
Bode diagram of closed-loop quadrature correction control circuits.

The transfer function of the closed-loop quadrature correction control system is:

(9) $$H_{qua}(s)=\frac{G(s,\omega_{d})K_{\text{int}}A_{x}\omega_{d}F_{LPF}(s)(K_{p}+K_{i}/s)}{1+G(s,\omega_{d})K_{\text{int}}A_{x}F_{LPF}(s)(K_{p}+K_{i}/s)k_{q}K_{ac}/2}$$

where $G(s,\omega_{d})=e^{j\theta }G(s-j\omega_{d})+e^{-j\theta }G(s+j\omega_{d})$, and $G(s)=1/(s^{2}+(\omega_{y}/Q_{y})s+\omega_{y}^{2})$. According to the simulation parameters in Table 4, the control system is simulated to optimize the control performance. The Bode diagram of closed-loop quadrature correction control circuits is shown in Figure 12. Obviously, a good closed-loop quadrature correction control is implemented.

## 5. Experiments

**Figure 13:**
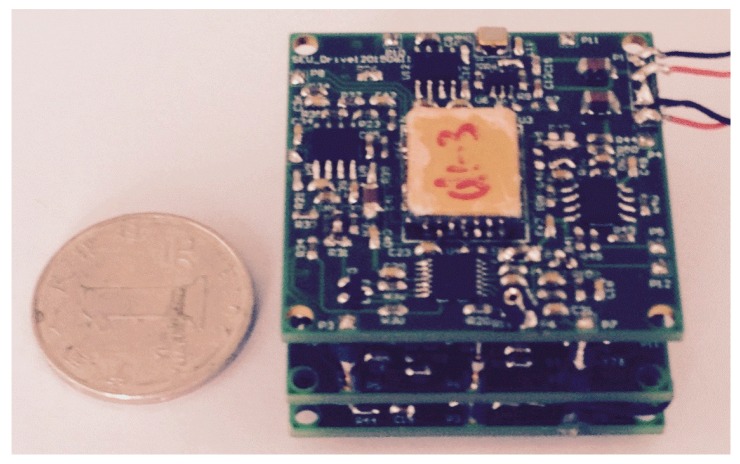
Prototype of DDMG.

**Figure 14:**
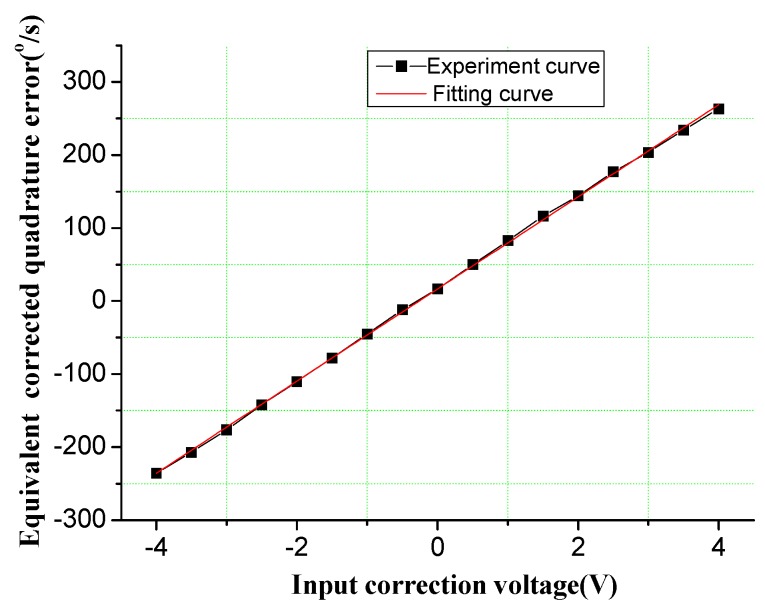
Correction capability experiment of quadrature error correction comb.

The structure of the DDMG shown in Figure 13 is packaged without vacuum encapsulation under standard atmospheric pressure. The quadrature correction mechanism is experimented to evaluate the correction capability of the quadrature correction comb. Results shown in Figure 14 demonstrate that the quadrature correction mechanism has a quadrature error correction capability of 63.06°/s/V. That is, when V = 5 V (see Equation (8)), an equivalent quadrature error of 63.06°/s can be suppressed completely by a feedback voltage of 1 V. Simultaneously, the quadrature correction system presents a slight nonlinearity in the large input correction voltage. The main reason is that the large correction force may cause the dynamic characteristic change of the gyroscope system.

**Figure 15:**
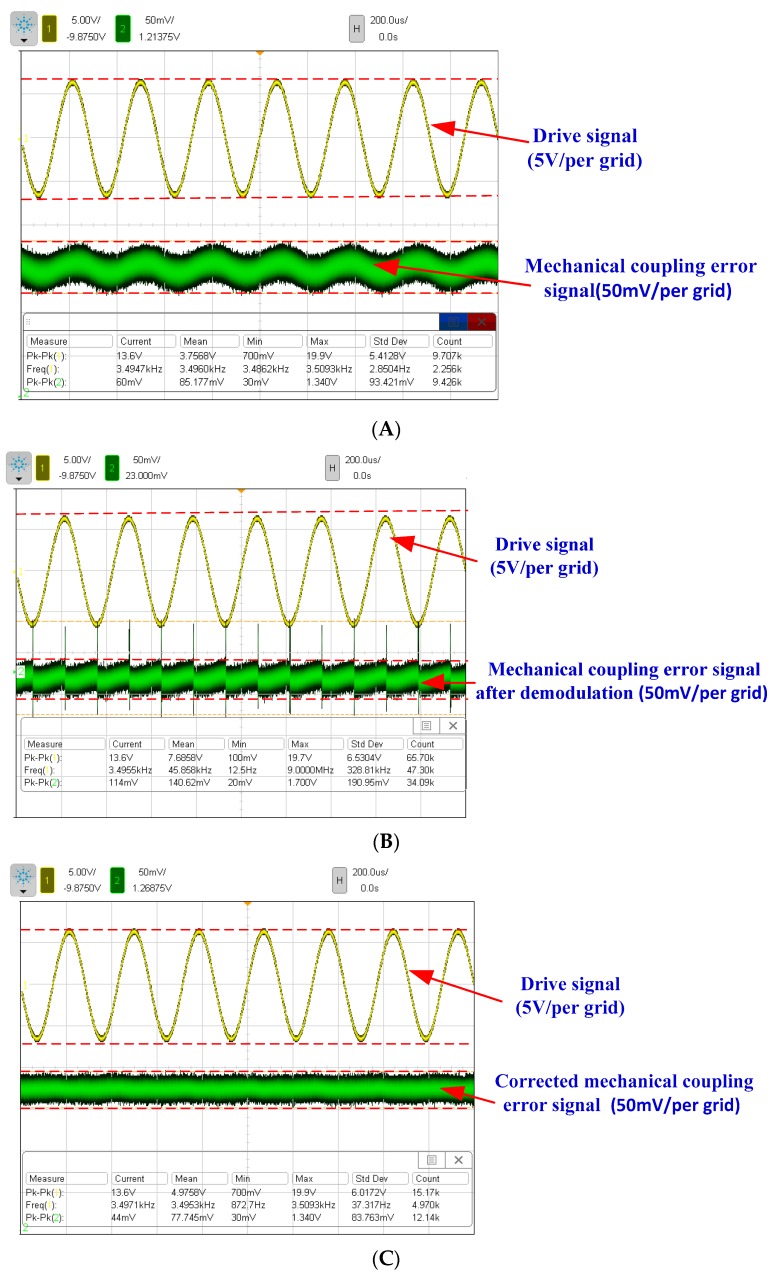
Mechanical coupling error measurement and suppression. (**A**) The mechanical coupling error signal waveform of the pre-amplifier in open loop sense (yellow signal is the reference drive signal in channel 1); (**B**) The mechanical coupling error signal waveform of the open loop sense after demodulation; (**C**) The mechanical coupling error signal waveform of the pre-amplifier after quadrature correction and feedback control.

The experiment is implemented to verify the suppression effect of the DDMG. Results of the mechanical coupling error measurement and suppression are shown in Figure 15. First, the mechanical coupling error signal waveform of the pre-amplifier in the open loop sense is shown in Figure 15A.

A mechanical coupling error signal with a peak-to-peak amplitude of 60 mV is measured. The demodulator is utilized to distinguish the quadrature error and bias error from the mechanical coupling error. According to the demodulated signal waveform shown in Figure 15B, the mechanical coupling error is substantially orthogonal with the reference demodulation signal. Apparently, most mechanical coupling errors are related to the quadrature error. Results demonstrate that the improved DDMG without vacuum encapsulation has a quadrature error of 16.43°/s and an offset error of 2.99°/s. Compared with the first-generation DDMG, the quadrature error in the improved DDMG is decreased by 9.66-fold (the original quadrature error in the first-generation micro-gyroscope is 158.65°/s), and the offset error is decreased by 6.36-fold (the original offset error in the first-generation micro-gyroscope is 19.03°/s). The optimized structure, which basically eliminates the rotational displacement errors by cancelling the moment arm of drive decoupled torque and suppresses a part of the translational movement displacement errors, significantly improves the suppression effect of the mechanical coupling error. However, the mechanical coupling errors cannot be completely eliminated because of fabrication error. The circuit control technologies shown in Figure 9 are introduced to further suppress the residual mechanical coupling error. In the quadrature control loop, the residual quadrature error is cancelled by the DC feedback voltages applied on the quadrature correction electrode. In the feedback control loop, the residual offset error is compensated by the modulated AC feedback voltages applied on the feedback electrode. Figure 15C shows the mechanical coupling error signal waveform of the pre-amplifier after quadrature correction and feedback control. The residual mechanical coupling error is substantially suppressed, which proves that the quadrature correction and feedback control circuits are feasible. Simultaneously, the quadrature error comparison with prior art is shown in Table 5. The quadrature error in this work is minimal. Ignoring the influence of fabrication error, the suppression of the non-ideal decoupling error provides the maximum possibility to reduce the mechanical coupling error.

**Figure 16:**
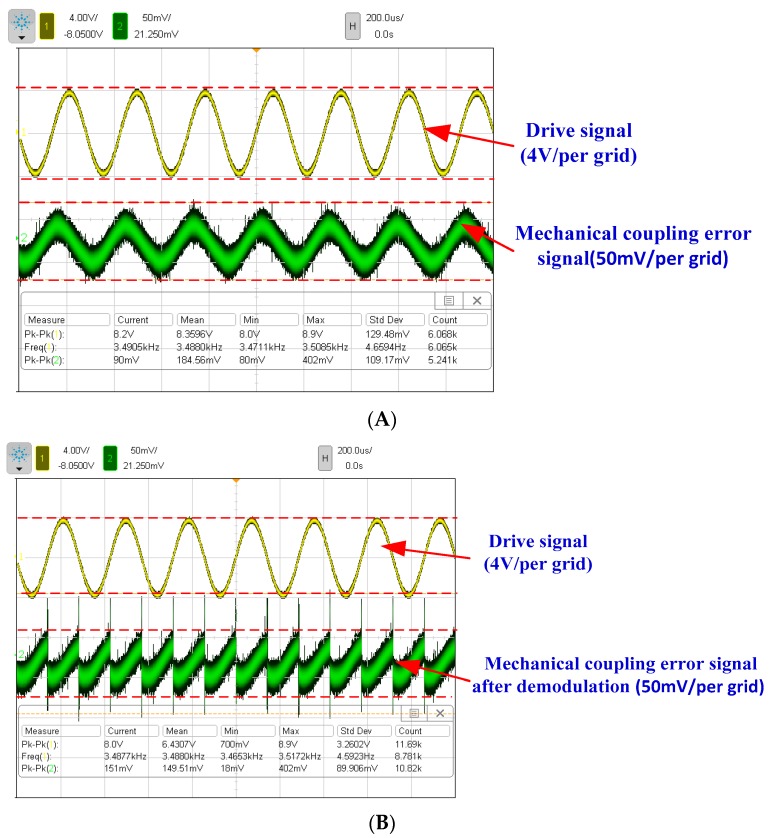
Mechanical coupling error measurement when the detection mechanism is exchanged with the driving mechanism. (**A**) The mechanical coupling error signal waveform of the pre-amplifier in the open loop sense (yellow signal is the reference drive signal in channel 1); (**B**) The mechanical coupling error signal waveform of the open loop sense after demodulation.

A comparison experiment between two kinds of decoupled beams in the same dual-mass micro-gyroscope structure is implemented to verify the suppression difference of mechanical coupling errors. Considering good interchangeability between the driving mechanism and detection mechanism, the detection mechanism is exchanged with the driving mechanism in the DDMG. The same driving force is applied on right and left proof masses by the detection mechanism along the sense direction. The original sense decoupled beam is utilized to decouple the driving movement in the sense direction. Furthermore, the original driving combs are used to measure the displacement of the mechanical coupling error. The mechanical coupling error signal waveform of the pre-amplifier is shown in Figure 16A. Similarly, the mechanical coupling error signal has a peak-to-peak amplitude of 90 mv. The demodulated signal waveform is shown in Figure 16B. The results demonstrate that the mechanical coupling error consists of a quadrature error of 31.23°/s and an offset error of 21.20°/s. With respect to the improved decoupling beam (drive decoupled beam), the original decoupling beam (the sense decoupled beam) leads to a 1.9-fold increase in quadrature error and 7.09-fold increase in offset error, which confirms that the improved design beam is effective.

**Figure 17:**
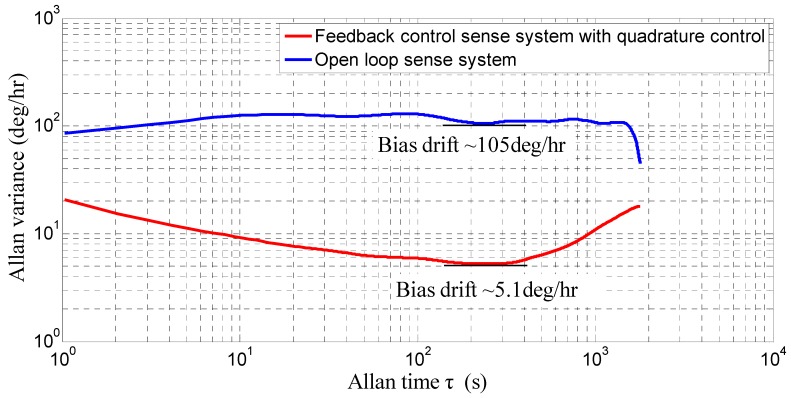
Bias drift in open loop sense system and feedback control sense system with quadrature control loop.

The DDMG prototype was analyzed to verify system performance. The open loop sense system and feedback control sense system with quadrature control loop were investigated. The bias stabilities in the open loop sense system and feedback control sense system with quadrature control loop are shown in Figure 17. Compared with open loop sense, the experiment results confirm that the feedback control sense system with the quadrature control loop decreases the bias drift of Allan variance by 20.59-fold, that is, the bias drift is decreased from 105°/h to 5.1°/h. The performance improvement is mainly due to the suppression of the mechanical coupling error. The suppression of the mechanical coupling error caused by the elimination of the non-ideal decoupling error can reduce the sensitivity of the circuit phase change on bias drift, which is beneficial to improve drift performance. Simultaneously, the closed-loop quadrature correction with feedback control further suppresses the residual mechanical coupling error and reduces the influence of system parameter variance on drift performance. Simultaneously, the input–output relationship between the open loop sense system and feedback control system is measured. With respect to the open loop sense, the feedback control sense system with quadrature control loop decreases the non-linearity of scale factor by 2.81-fold in the ±400°/s range, that is, the non-linearity of scale factor is decreased from 234.6 ppm to 83.5 ppm. The main reason is that the feedback control system always restores the proof mass to the equilibrium regardless of the angular velocity input, which is beneficial to improve the non-linearity of scale factor.

## 6. Conclusions

An improved DDMG for mechanical coupling error suppression is presented in the paper. The structure principle and the suppression of quadrature error of DDMG are described in detail. The structure simulation of the micro-gyroscope is achieved to confirm the suppression effect of the mechanical coupling error. Compared with the former structure, the rotational displacements of the improved structure in the sense frame are substantially suppressed in the drive mode, and the translational movement displacements of the improved structure in the sense frame are reduced by one in the drive mode. We adopt the DDSOG process to fabricate the DDMG structure chip. The quadrature correction and feedback control circuits are designed. Finally, a DDMG prototype is tested to verify the system performance. Compared with the former dual-mass micro-gyroscope, the quadrature error in the improved DDMG is decreased by 9.66-fold, and the offset error is decreased by 6.36-fold. Compared with the open loop sense, the feedback control circuits with quadrature control loop decrease bias drift by 20.59-fold and decrease the scale factor non-linearity by 2.81-fold in the ±400°/s range.

## Figures and Tables

**Table 1:** Simulation parameters.

Parameter		Value	Parameter	Value
Length × Width (µm)	Drive decoupled beam	617 × 10	Thickness *h* (µm)	60
	Drive suspension beam	630 × 10	Single proof mass *m* (kg)	5.13 × 10^−7^
	Sense suspension beam	518 × 10	Drive mode Q-factor *Q_x_*	400
	Sense decoupled beam	540 × 10	Sense mode Q-factor *Q_y_*	255
	Suspension beam of frame	540 × 10	Single drive stiffness *k*_x_ (N/m)	205.6
	Sense coupling suspension beam	665 × 10	Drive coupling stiffness *k*_ox_ (N/m)	32.5
	Drive coupling suspension beam	648 × 10	Single sense stiffness *k*_y_ (N/m)	247.4
	Lever support beam	375 × 10	Sense coupling stiffness *k*_oy_ (N/m)	9.1

**Table 2:** First fifth modes of the DDMG.

Modal	1	2	3	4	5
Frequency (Hz)	3157	3463	3588	3622	6912

**Table 3:** Non-ideal decoupled displacement in the drive and sense modes.

	Measurement Point	Non-Ideal Decoupling Displacement			
		Original Structure		Improved Structure	
		*X*-axis	*Y*-axis	*X*-axis	*Y*-axis
Drive mode	A	8.2355	−25.697	−4.18045	−0.0142
	B	3.56	0.2235	−4.2411	0.08024
	C	8.2355	26.164	−4.18135	−0.0143
	D	959.71	0.1766	−960.14	0.08366
Sense mode	E	−7.5332	−12.260	10.093	−1.4942
	F	0.1549	−12.112	−0.11313	−1.5029
	G	7.8458	−12.260	−10.319	−1.4942
	H	0.1603	−900.11	−0.11848	−909.05

**Table 4:** Simulation parameters.

Parameter	Value	Parameter	Value
ω*_d_* (rad/s)	3588 × 2π	n_q_	34
ω*_y_* (rad/s)	3622 × 2π	*k_q_*(N/(m·V·kg))	44,054
*A_x_* (µm)	5	*K_p_*	1
*K_ac_*	162.5	*K_i_*	100
*V* (V)	5	*Q_y_*	200
*h* (µm)	60	*K_int_*	640,000
d_ss_ (µm)	4	F_LPF_(s)	20,000/(s^2^ + 150s + 20,000)

**Table 5:** Quadrature error comparison with prior art.

Reference		Parameter	
	Quadrature error(°/s)	Decoupling mechanism	Proof mass amount
[17]	300	Part- decoupling	Single
[18]	2000	Part-decoupling	Dual
[19]	75	Whole-decoupling	Dual
[20]	500	Whole-decoupling	Dual
[13]	158.65	Whole-decoupling	Dual
This work	16.43	Whole-decoupling	Dual
